# Goldmann and error correcting tonometry prisms compared to intracameral pressure

**DOI:** 10.1186/s12886-017-0668-z

**Published:** 2018-01-04

**Authors:** Sean McCafferty, Jason Levine, Jim Schwiegerling, Eniko T. Enikov

**Affiliations:** 1 Department of Ophthalmology, Intuor Technologies, University of Arizona- College of Medicine, University of Arizona- College of Optical Science, LLC 6422 E. Speedway Blvd. Suite 100, Tucson, AZ 85710 USA; 2 Department of Ophthalmology, University of Arizona- College of Medicine, 6422 E. Speedway Blvd. Suite 100, Tucson, AZ 85710 USA; 3 Department of Ophthalmology, University of Arizona-College of Optical Science, University of Arizona-College of Medicine, 1630 E. University Blvd, Tucson, AZ 85719 USA; 4 Department of Aerospace and Mechanical, University of Arizona-College of Engineering, 1130 N. Mountain Ave, Tucson, AZ 85721 USA; 5 Tucson, USA

**Keywords:** Glaucoma, Intraocular pressure, IOP, Goldmann, Bias, Error, Perkins, Tonometer, Applanation, CCT, Central corneal thickness, CRF, Corneal resistance factor, Intracameral, Cadaver eye, In vivo, In vitro, Head position, Upright, Supine, Manometric, Corneal hydration

## Abstract

**Background:**

Compare Goldmann applanation tonometer (GAT) prism and correcting applanation tonometry surface (CATS) prism to intracameral intraocular pressure (IOP), in vivo and in vitro.

**Methods:**

Pressure transducer intracameral IOP was measured on fifty-eight (58) eyes undergoing cataract surgery and the IOP was modulated manometrically to 10, 20, and 40 mmHg. Simultaneously, IOP was measured using a Perkins tonometer with a standard GAT prism and a CATS prism at each of the intracameral pressures. Statistical comparison was made between true intracameral pressures and the two prism measurements. Differences between the two prism measurements were correlated to central corneal thickness (CCT) and corneal resistance factor (CRF). Human cadaver eyes were used to assess measurement repeatability.

**Results:**

The CATS tonometer prism measured closer to true intracameral IOP than the GAT prism by 1.7+/−2.7 mmHg across all pressures and corneal properties. The difference in CATS and GAT measurements was greater in thin CCT corneas (2.7+/−1.9 mmHg) and low resistance (CRF) corneas (2.8+/−2.1 mmHg). The difference in prisms was negligible at high CCT and CRF values. No difference was seen in measurement repeatability between the two prisms.

**Conclusion:**

A CATS prism in Goldmann tonometer armatures significantly improve the accuracy of IOP measurement compared to true intracameral pressure across a physiologic range of IOP values. The CATS prism is significantly more accurate compared to the GAT prism in thin and less rigid corneas. The in vivo intracameral study validates mathematical models and clinical findings in IOP measurement between the GAT and CATS prisms.

## What was known

1. Overall bias and biomechanical errors in Goldmann tonometry exist and have been demonstrated comparing to true intracameral IOP.
2. Patient positional errors exist in GAT IOP measurement, and have been quantified.

## What this paper demonstrates

1. Quantifies statistically the overall decrease in error in the CATS prism compared to the GAT prism in relation to true intracameral IOP.
2. Live human eye manometric adjustment and maintenance of intracameral IOP at three (3) separate physiological values comparing GAT and CATS prism measured IOP
3. Demonstrates effect of CCT and CRF measured values in live human eyes and correlates measured IOP error to both GAT and CATS prism use.
4. Examines statistical variation and repeatability of IOP measurements between the GAT and CATS prism in human cadaver eyes.

## Background

Goldmann Applanation Tonometry (GAT) to measure Intraocular pressure (IOP) today remains the gold standard. It is the primary metric in the diagnosis and treatment of glaucoma as well as many other pressure related processes [1–4]. Central corneal thickness (CCT) correction is the only common clinical correction for the several identified GAT IOP corneal biomechanical measurement errors [5–10]. CCT IOP error correction has been shown to be inadequate by itself [11, 12]. The GAT IOP measurement in comparison to true intracameral IOP measured by pressure transducer has been shown to have significant underestimating bias [10, 13–16]. Direct correlations between GAT IOP error and corneal biomechanical parameters have been demonstrated relative to intracameral pressure [8, 10, 13–16]. Previous studies have demonstrated overall bias in GAT IOP measurement as well as errors due to patient specific biomechanical parameters and patient position [10]. In addition, two previous studies have examined both the theoretical modeling and direct clinical comparison of a modified applanation surface GAT (or CATS) prism to the traditional flat GAT prism [9, 17]. Both have demonstrated decreased CATS sensitivity to corneal biomechanical error parameters. The present clinical study was designed to compare both a GAT prism and a modified applanation surface (CATS) prism in live human subjects to a true ‘gold standard’ intracameral pressure which was manometrically adjusted over the physiologic range of IOP measuring bias and sensitivity to corneal biomechanical parameters. Additionally, human cadaveric eye testing was completed to determine Inter-operator and intra-operator measurement repeatability error comparison between the CATS and GAT prisms.

## Methods

The correcting applanation tonometry surface (CATS) tonometer prism is a modified GAT prism which optimizes the corneal applanating surface to decrease the sensitivity of applanation tonometry to corneal biomechanical variability [17]. The CATS prism is an investigational device and has not been approved for clinical use. The CATS prism is designed to be a replacement prism for any existing Goldmann or Perkins tonometer. Measurement technique of the CATS prism and the force to pressure conversion is unchanged from the GAT prism.

**Fig. 1:**
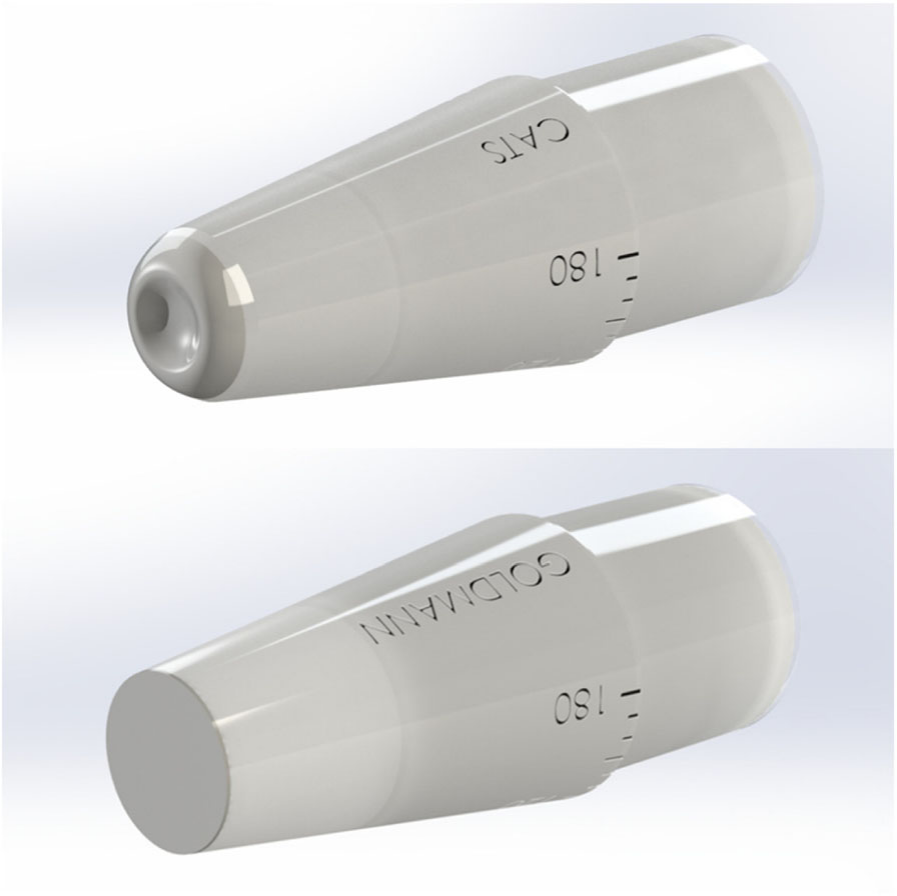
CATS tonometer prism with modified applanating surface versus GAT

In mathematical modeling, the CATS tonometer prism, illustrated in Fig. 1, reduces GAT measurement error due to recognized variations in corneal biomechanics by approximately 50% [17]. The reductions in error due to biomechanical parameters were also validated in a clinical study examining the direct difference in IOP measurement between CATS and GAT prisms when correlated to measured biomechanical parameters [9].

### Human surgical eye testing (in vivo study)

A prospective intra-surgical clinical study was performed at Carondelet Foothills Ambulatory Surgery Center in Tucson, Arizona. Fifty eight (58) eyes (from 48 patients) aged 18 and older and were enrolled from the Arizona Eye Consultants clinic. A sample size of fifty eight (58) eyes was determined sufficient to demonstrate statistical correlation from previous studies [13–15, 17, 18]. The prospective study enrolled patients scheduled for phacoemulsification, cataract surgery. A thorough ophthalmic exam was completed on all patients by one of two licensed investigators (SM, JL) to include slit-lamp biomicroscopy, anterior segment ocular coherence tomography (OCT) with central corneal thickness (CCT measurement (Zeiss HD-OCT, Jena, Germany), corneal topography (Zeiss Atlas model 9000 Jena, Germany), dilated funduscopy and an Ocular Response Analyzer (ORA) with corneal resistance factor (CRF) derived from corneal hysteresis (CH) measurements (Reichert Ophthalmic Instruments, Depew, New York). The study enrollment criteria included: (1) clinical indications for phacoemulsification (2) adequate patient target fixation (3) corneal curvature between 38.00 and 50.00 diopters (D); and (4) Less than 3.50 D of corneal astigmatism. Subjects were selected in accordance with the following exclusion criteria: Ocular surgery within the last 3 months; pregnant or nursing: only one functional eye; poor or eccentric fixation; high corneal astigmatism (>3.5 diopters); corneal scarring; corneal surgery; microphthalmos; buphthalmos; severe dry eyes; blepharospasm; nystagmus; keratoconus; or any other corneal or conjunctival pathology or infection.

The research protocol conformed to the tenets of the Helsinki Declaration and was approved by Chesapeake Independent Review Board (IRB). All patients received a complete informed consent detailing risks of the study verbally and in writing.

Measurements were performed in the following order: CCT, topography, ORA, Applanation IOP with intracameral IOP. Each investigator was masked to the results of the other tests. Anterior segment OCT with CCT, corneal topography, and ORA with CRF were measured by a non-surgical investigator 1 day before surgery. With a spectral domain ocular coherence tomographer HD-OCT, the corneal thickness at 3 locations was measured and averaged for analysis.

Corneal biomechanical properties were approximated by measurements with an ORA by a non-surgical investigator 1 day before surgery. Topical anesthetic drops were applied so that examination conditions were equivalent to other measurements in this study. CRF was measured as an indicator of corneal biomechanical properties. CH results from the dynamic nature of the air pulse and the viscous damping inherent in the cornea. It was measured as the difference between the inward (P1) and the outward (P2) applanation pressures. CRF is an empirically derived measurement from CH of both the viscous and elastic resistance encountered by the air jet while deforming the corneal surface. It is equal to (P1–0.7P2) [6, 8]. ORA measurements were taken in triplicate, and the average value was taken for statistical analysis. Off-scale values were discarded, as well as measurements that could not be repeated three times. A Zeiss HD-OCT-5000 spectral domain ocular coherence tomographer was used by the assistant to measure central corneal thickness. Finally, the assistant investigator completed a corneal topography and an averaged corneal curvature was used for analysis over the central 3 mm diameter of the cornea in accordance with ANSI Z80.23. The surgical investigator conducting IOP measurements was masked to the results of the assistant investigator’s tests.

A standard surgical prep and drape was completed followed by the initial surgical ocular incisions. Intracameral preservative-free lidocaine 1% (1cm^3^) was instilled in the anterior chamber. At this point, the disposable anterior chamber cannula (Sterimedix, Reddich, UK) was placed through the surgical paracentesis and checked to insure no leaks were present around the cannula. The Incision was 1.2 mm at a ‘near clear’ corneal location almost tangential to the limbus. The cannula and tubing were adjusted and secured throughout the measurements to eliminate any visible endothelial folds minimizing potential changes to the biomechanical properties of the central cornea. Surgical Balanced Salt Solution (BSS) was used to maintain and adjust the anterior chamber pressure by elevating bottle height (Alcon, Ft. Worth, TX). The intracameral surgical tubing was attached to a disposable right heart catheter pressure tranducer (Transpac IV, ICUMedical, San Clemente, CA)(accuracy +/−1%) and zeroed through the monitor (DatexOmeda S/5, Ge Healthcare, Chicago, Il) at a bottle height level with the anterior chamber of the surgical eye. Pressure Data was recorded at 25 Hz on S/5 Collect software (Ge Healthcare, Chicago, Il). Intracameral IOP was adjusted and allowed to stabilize at 10 mmHg as measured by the pressure transducer. Tear film was standardized by using Weck-cell sponge drying of the ocular fornices prior to measurement. A sterilized and calibrated Perkins type GAT tonometer was then used by the surgical investigator to measure applanation IOP at two averaged measurements each with the Perkins tonometer. Fluorescein (Fluorescein Sodium Ophthalmic Solution 0.25%/0.4%, Bausch & Lomb, Tampa, FL) was applied prior to each measurement so that examination conditions were equivalent. Measurements of IOP were made two (2) times with the Perkins tonometer (one measurement was considered by averaging measurements at 180 and 90 degrees to correct for astigmatism). If the sequential measurements with one prism were more than 2 mmHg different, then a third measurement was obtained. All three measurements were then averaged. The third measurements were included in the study if it was within the range of the first two, otherwise all measurements were discarded. The intracameral IOP was then adjusted and allowed to stabilize at 20 mm and 40 mmHg as measured by the pressure transducer and the IOP measurement was repeated with the Perkins tonometer.

Statistical analysis included pressure comparisons between the CATS and GAT prisms to true intracameral pressure noting the average and standard deviation with Homeoscadastic two-tailed Student’s-t test to examine probable significance of the differences. Reported values for tonometer measured IOP were corrected for the upright applanation tonometry position in order to be most applicable to typical clinical conditions. This correction of 2.7 mmHg in vivo was validated in our previous publication examining overall and positional error in applanation tonometry [10]. Linear correlation coefficients were examined with the CATS and GAT prism IOP measurements versus measured error parameters of CCT and CRF. A multivariate regression analysis with a linear mixed-effects model was carried out to compare sensitivities of the GAT and CATS IOP reading errors to CCT and CRF. Separately, the differences in CATS and GAT measurements were examined in thin corneas (CCT < 530 μm) and thick corneas (CCT > 570 μm) with Student’s-t test for probable significance.

### Human cadaveric eye testing (in vitro study)

Cadaveric eye testing was completed human globes to determine practitioner intra-operator and inter-operator repeatability of pressure measurements with both the CATS and GAT prisms. Twenty one (21) enucleated human globes were obtained from the Georgia Eye Bank (Atlanta, GA). The whole globes were shipped less than 24 h post-mortem and stored at 4 °C in Optisol chambers until use [18]. All corneas were of corneal transplant quality without prior surgery. The cadaver eyes are used on the day of arrival within 36 h post mortem. The eyes, ages of the cadavers, and cause of death were recorded. Eyes with a history or evidence of previous anterior segment intraocular surgery (except cataract) or corneal abnormalities were excluded.

**Fig. 2:**
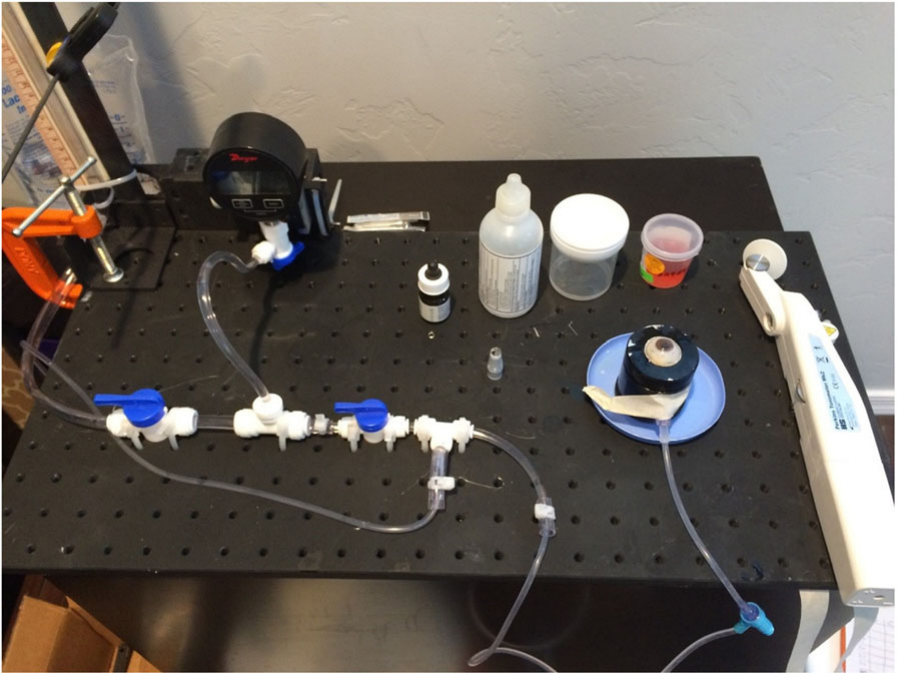
Ocular globe IOP apparatus for measuring IOP in the supine position, showing a Perkins type tonometer

They were stabilized in a specially designed apparatus for manometrically pressurizing and measuring IOP in a whole globe (Fig. 2) with the cornea exposed.

Standard biological precautions were followed when handling eye tissue. The corneal thickness was measured via Reichert pachymeter for IOP correlation to corneal thickness errors. The corneal thickness at central location was measured 3 times and averaged for analysis.

**Fig. 3:**
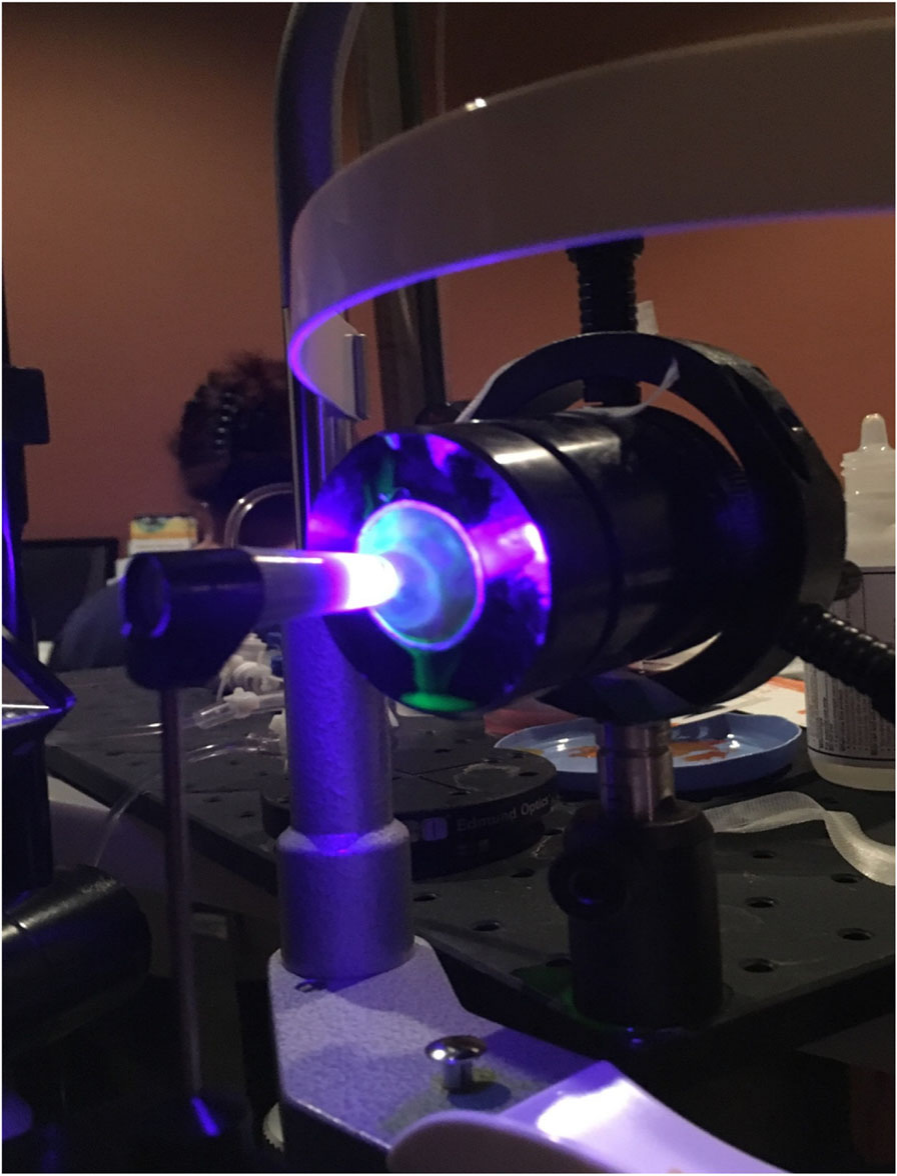
Ocular globe IOP apparatus for measuring upright IOP showing Goldmann type tonometer

All 21 eyes remained epithelized and hydrated with standard isotonic BSS. BSS was used to hydrate the corneal epithelium between measurements before the application of fluorescein solution. A 22-gauge needle with Y-adaptor (Saf-T-Intima, Vialon; Becton, Dickinson and Company, Franklin Lakes, NJ) was then inserted into the anterior chamber via a separate scleral approach. Extreme care was taken with all penetrations of the eye to avoid touching the endothelium, the iris, or the lens. The entire globe was mounted in the eye stabilization device shown in Fig. 2 embedded in moisturized gauze. Subsequently, the IOP was measured at the set manometric pressure in the upright position with the Slit-lamp mounted Goldmann tonometer H-S 900 (Fig. 3). The globe elevation at the central cornea was maintained equal in all measurements to insure a constant intracameral IOP. IOP measurements were completed only at a single intracameral pressure for each globe [18]. The needle IV tube was connected to a manometric transducer (Dwyer Instruments, Michigan City, IN), an isotonic sodium chloride solution infusion bottle, and an open-air reference tube.

Multiple stopcocks were attached to bleed all bubbles from the system and to allow either open or closed stopcock techniques. The transducer and the anterior chamber were maintained at the same height for all measurements. The isotonic sodium chloride solution infusion bottle was attached to a manually driven intravenous pole for bottle height adjustment.

IOP measurements were taken utilizing a slit lamp mounted GAT for upright measurements [19]. Twenty one (21) total cadaver eyes were utilized. The eyes were measured at each of the following seven (7) intracameral pressures (5, 10, 20, 30, 40, 50, 60 mmHg). Measurements were completed five (5) times by two (2) different examiners (10 total) with each prism. Each measurement consisted of a standard reference axis measurement averaged with a measurement rotated counter-clockwise 90 degrees from the standard reference axis to account for any astigmatic errors. A randomization occurred to determine which prism was utilized first. BSS was used in the application of fluorescein solution to limit epithelial toxicity. After each series of measurements on an eye at a given pressure, the bottle height was lowered to the initial 4.8 cm. The series was only accepted if the initial and closing manometric pressures were within ±1 mmHg.

Statistical analysis included pressure comparisons between the CATS and GAT prisms to true intracameral pressure noting the average and variance. Homeoscadastic two-tailed Student’s-t test was used to examine probable significance of the differences between individual operators and overall differences in IOP measurement between the CATS and GAT prisms.

## Results

**Fig. 4:**
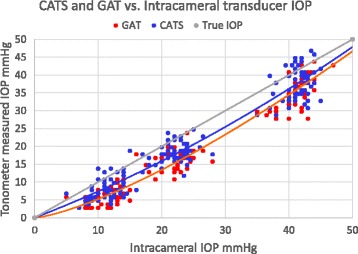
In Vivo Perkins IOP measurement scatterplot using the CATS and GAT prisms over all Intracameral IOPs In live human eyes undergoing cataract surgery

Intraocular pressure measurements using the Perkins applanation tonometer and the cannulated transducer IOP on patients undergoing cataract surgery were completed on 58 eyes of 48 patients. The study’s average subject age was 66+/−8 years with 31 females and 27 males. The Perkins applanation IOP measured in the supine position was significantly less than the Intracameral transducer measured pressure at all three modulated pressures (10, 20, and 40 mmHg). See Fig. 4 illustrating the measured applantion IOP lines using both the CATS and GAT prisms under the true intracameral IOP line. The lines demonstrating the differences in IOP measurement of the CATS and GAT prisms compared to intracameral pressure are a closest fit polynomial forced through zero. The zero intercept is justified since the prisms are unable to measure a negative pressure and would only read zero.

**Fig. 5:**
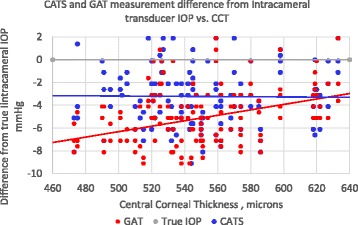
CATS and GAT IOP measurement difference from true intracameral IOP correlated to central corneal thickness (CCT)

The Intracameral pressure referenced error in CATS and GAT prisms was measured and correlated to central corneal thickness (CCT). The subject’s average CCT was 548+/− 40 μm which is comparable to a similar study at 556+/−40 μm [11]. Figure 5 illustrates decreased slope sensitivity to CCT and increased accuracy CATS prism in thin corneas (CCT < 530 μm). The CCT sensitivity slope is reduced from 0.024 mmHg/μmCCT with the GAT to −0.0006 mmHg/μmCCT with the CATS prism. In thick corneas, the added force required to applanate the cornea with the GAT prism negates much of the measurement difference between the CATS and GAT (CCT > 600 μm). A multivariate regression analysis with linear mixed-effects revealed a statistically significant (*p* = 0.021) difference in sensitivity to CCT between the GAT and CATS.

**Fig. 6:**
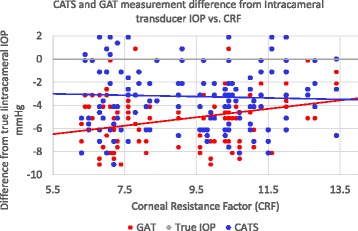
CATS and GAT IOP measurement difference from true intracameral IOP correlated to corneal resistance factor (CRF)

The Intracameral pressure referenced error in CATS and GAT prisms was measured and correlated to ORA measured CRF. The subject’s average CRF was 9.2 +/−2.1. Figure 6 illustrates the decreased slope sensitivity to CRF in the CATS prism. It demonstrates a linear error sensitivity of 0.37 mmHg/CRFunit with the GAT and −0.043 mmHg/CRFunit with the CATS prism which is nearly statistically significant in the in the linear mixed effects analysis when compared to the GAT (*p* = 0.055). The added force required to applanate the cornea with the GAT prism in ridged or high CRF (>11.0 units) subjects negates much of the measurement difference between the CATS and GAT. The low CRF corneas (<8.5 CRF units) are more accurate compared to intracameral pressure in the CATS prism by 2.8+/−2.1 mmHg which is statistically significant (p = 0.05).

**Fig. 7:**
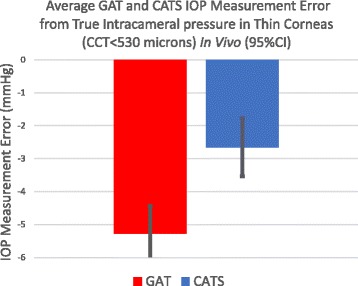
Average CATS and GAT IOP measurement error from true intracameral pressure in thin corneas (CCT < 530 μm)

**Fig. 8:**
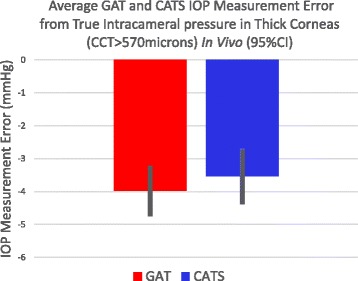
Average CATS and GAT IOP measurement error from true intracameral pressure in thick corneas (CCT > 570 μm)

The statistical significance of IOP measurement error was also separately calculated in both thin (<530 μm) and thick (>570 μm) corneas. Figure 7 indicates a statistically significant improvement in IOP measurement accuracy compared to intracameral pressure of 2.7+/−1.9 mmHg in subjects with CCTs under 530 μm (*p* = 0.01). Figure 8 shows a statistical equivalence (0.5+/−2.2 mmHg) in IOP measurements between the CATS and GAT prisms in thick corneas with CCTs over 570 μm (*p* = 0.33).

**Fig. 9:**
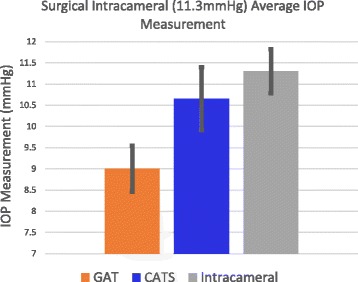
CATS and GAT IOP measurements compared to an average true intracameral pressure of 11.3 mmHg

**Fig. 10:**
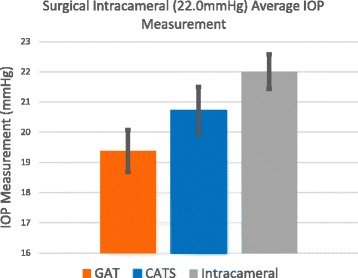
CATS and GAT IOP measurements compared to an average true intracameral pressure of 22.0 mmHg

Figures 5 and 6 show an IOP measurement bias error between the CATS and GAT prisms of 1.70+/−2.74 mmHg which is statistically significant (*p* = 0.04). However, if the low intracameral pressures at 10 mmHg intracameral pressure are removed and only the pressures at 20 and 40 mmHg are examined the calculated bias of 1.23+/−3.19 mmHg is not statistically significant (*p* = 0.18). The low pressure improved accuracy of the CATS prism is shown in the comparison of IOP measurement errors at an average of 11.3 mmHg intracameral pressure in Fig. 9 and average of 22.0 mmHg in Fig. 10. The improved accuracy at low IOP (<10 mmHg) is also demonstrated in the cadaver testing below.

The variability of the plots in Figs. 5 and 6 is indicative of multiple competing corneal biomechanical properties as well as testing errors affecting the pressure measurement. The multivariate regression analysis with linear mixed-effects revealed a statistically significant (*p* = 0.021) difference in sensitivity to CCT and a nearly statistically significant (*p* = 0.055) difference in sensitivity to CRF between the GAT and CATS. A post-hoc power calculation of the 58 eyes on 48 patients was completed and found to be 88.5% (alpha = 0.05). However, even if we consider zero independence between contralateral eye measurements with 48 patients our post-hoc power calculation remains high at 75.0%.

**Fig. 11:**
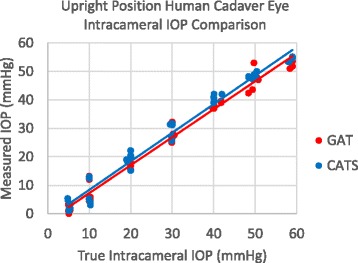
CATS and GAT prism cadaver IOP measurement comparison to intracameral pressure in human cadaver eyes

Twenty one (21) human cadaver eyes were measured and analyzed each at a singular intracameral pressure. The average age of the donor was 59+/−19 years with 17 male and 4 female. The CATS and GAT prisms were randomized and used to measure IOP in a Goldmann tonometer in the upright position. Figure 11 shows Intraocular pressure measurement in cadaver eyes over all pressures. There was no significant difference between the CATS and GAT prisms with all pressures included (*p* = 0.19).

Intra-operator repeatability is illustrated in Table 1. The differences in repeatability as measured by the coefficient of variation are not statistically significant between the CATS and GAT prisms with all pressures combined. The accuracy of the CATS prism compared to the GAT prism approaches a significant improvement at the low pressure of 5 mmHg (*p* = 0.07) and at 40 mmHg (*p* = 0.05). Inter-operator repeatability was examined between the two masked practitioners measuring IOP alternately and there was no statistical IOP error difference between the two operators using the CATS or GAT prisms (*p* = 0.40,0.32, respectively).

**Table 1:** IOP measurement variance and accuracy with the CATS and GAT prisms in cadaver eyes

Overall accuracy/repeatability	Parameter	5 mmHg	10 mmHg	20 mmHg	30 mmHg	40 mmHg	50 mmHg	60 mmHg
GAT reference prism	Mean IOP meas.	1.1	7.6	17.7	27.8	38.0	47.0	54.1
	Std. Dev.	1.2	3.6	1.5	2.2	1.1	3.6	2.6
	Coeff. of Variation	1.1	0.5	0.1	0.1	0.0	0.1	0.0
	Accuracy (error to transducer IOP)	−4.0	−2.5	−2.3	−2.3	−2.6	−2.5	−5.5
CATS prism	Mean IOP meas.	3.0	7.1	19.2	29.0	41.1	48.4	55.0
	Std. Dev.	1.7	4.0	2.2	2.6	1.2	1.2	1.6
	Coeff. of Variation	0.6	0.6	0.1	0.1	0.0	0.0	0.0
	Accuracy (error to transducer IOP)	−2.1	−3.0	−0.5	−0.9	0.4	−1.1	−4.3

## Discussion

The In vivo (surgical eye study) results demonstrated a statistically significant decreased CATS prism sensitivity in IOP measurement error due to patient variability in corneal thickness (CCT) and nearly statistically significant decreased sensitivity to corneal rigidity (CRF) when compared to the GAT prism. The decreased error parameter sensitivity results in a statistically improved accuracy in IOP measurement compared to true intracameral pressure with the CATS prism in patients with relatively thin corneas (<530 μm). The added force required to applanate the cornea with the GAT prism in high CCT (>570 μm) or ridged CRF (>11.0 units) subjects negates much of the measurement difference between the CATS and GAT. The results verify the previously published mathematical modeling and the expected slope in the difference between CATS and GAT measurements when correlated to each of the error parameters of corneal thickness, corneal rigidity, and corneal curvature [17]. Additionally, a clinical study of 109 eyes correlating the expected slope correction difference between CATS and GAT IOP measurements to corneal biomechanics related errors corroborate the present findings of decreased sensitivity [9]. The aforementioned study did not compare IOP to intracameral pressures and were measured in a narrow pressure range of 17.5+/−2.8 mmHg. The 109 eye clinical study indicated zero bias error between the CATS and GAT prisms. Low correlation coefficients are common in clinical IOP studies due to the multiple variables in measurement error [20–23]. The assumption made in statistical analysis examining correlation is that the biomechanical error relationships are linear when in fact there is evidence that they may be non-linear which may add to a lower correlation [24]. About 30 % (30.8%) of a standard patient population with CCT measurements under 530 μm will have an under-estimation of IOP by 2.7 mmHg using the GAT prism compared to the CATS prism. This GAT prism underestimation error is increased to an average of 3.7 mmHg compared to the CATS prism when the CCT is less than 500 μm. The present GAT CCT error findings are consistent with previous studies verifying the Dresdner CCT correction [24]. It has been demonstrated that the CATS prism has the capacity to correct for more than CCT in that it also corrects for corneal rigidity, curvature, and tear film adhesion [17]. The average CCT correction difference between the CATS and GAT may be significantly higher as many of the patients with CCTs under 530 μm also have rigid and steep curvature corneas which reduce the correlation slope due to the multiple competing variables creating IOP measurement error.

The CATS and GAT prisms statistically measure the same IOP on average with the exception of low pressures (<10 mmHg) in which the CATS prism measures significantly more accurately by 18.9% when compared to true intracameral pressure. The CATS prism’s low pressure improved accuracy was seen with both in vivo and in vitro testing. The CATS prism was designed to measure the same as the GAT for a nominal cornea with average biomechanical properties. The GAT prism was shown to have significant overall bias error to intracameral pressure [10]. The CATS prism could significantly negate the bias error in GAT IOP measurement as it was first designed. However, the CATS prism was subsequently re-designed to roughly maintain the same amount of overall bias error thus not requiring the clinician to readjust long standing benchmarks considered as low, average and high IOP. It is possible that the original overall bias negating design could be useful in pediatric, post-corneal refractive and veterinary applications where bias errors are likely more prominent [15, 18, 25].

Human cadaver eye IOP measurements were statistically equivalent between the CATS and GAT prisms over all measured IOPs. No significant bias error was noted at each intracameral adjusted pressure, except at the low pressure of 5 mmHg and at 40 mmHg. Cadaver eye correlations to CCT and CRF are difficult as they change rapidly post-mortem and are as much a factor of the time since death with associated corneal hydration as any property of the cornea while the subject was alive.

Both CATS and GAT applanation tonometry prisms require a centered cornea on the prism face to accurately measure IOP. Although centration is required with the GAT for accurate measurement, the GAT prism will measure applanated mires imaged through the prism anywhere on the flat prism face. The CATS tonometer prism’s concave-convex surface does not allow the mires to intersect unless the prism is centered on the cornea. Repeatability in CATS prism IOP measurements are shown in the cadaver eye analysis to be the same as the existing GAT reference prism, both in serial repeat IOP measurements and between two masked practitioners.

Corneal curvature was not considered in the correlations as this can be altered significantly under surgical conditions being supine and having an anterior chamber cannula placed for pressure monitoring. However, care was taken to standardize the incision location and cannula position to minimize alterations in corneal stress and deformation.

Clinicians today almost universally have the capability to measure IOP with a GAT, and a majority of practitioners consider it the most accurate measurement of IOP. GAT errors are well known to most clinicians, and current clinical practice does not correct for most corneal biomechanical errors. However, the CATS tonometer demonstrates the capacity to correct for these inaccuracies and can provide a single error-corrected IOP without additional measurement, calculations, or interpretation error.

## Abbreviations

BSS: Balanced salt solution
CATS: Correcting applanation tonometry surface
CCT: Central corneal thickness
CH: Corneal hysteresis
CRF: Corneal resistance factor
GAT: Goldmann applanation tonometer
IOP: Intraocular Pressure
OCT: Optical coherence tomography
ORA: Ocular response analyzer
